# The Role of Adjunctive Therapies in Septic Shock by Gram Negative MDR/XDR Infections

**DOI:** 10.1155/2017/2808203

**Published:** 2017-07-09

**Authors:** Stefano Busani, Erika Roat, Giulia Serafini, Elena Mantovani, Emanuela Biagioni, Massimo Girardis

**Affiliations:** Intensive Care Unit, Modena University Hospital, L.go del Pozzo 71, 41100 Modena, Italy

## Abstract

Patients with septic shock by multidrug resistant microorganisms (MDR) are a specific sepsis population with a high mortality risk. The exposure to an initial inappropriate empiric antibiotic therapy has been considered responsible for the increased mortality, although other factors such as immune-paralysis seem to play a pivotal role. Therefore, beyond conventional early antibiotic therapy and fluid resuscitation, this population may benefit from the use of alternative strategies aimed at supporting the immune system. In this review we present an overview of the relationship between MDR infections and immune response and focus on the rationale and the clinical data available on the possible adjunctive immunotherapies, including blood purification techniques and different pharmacological approaches.

## 1. Introduction

Since early 90s, the American College of Chest Physicians and Society of Critical Care Medicine Consensus Conference has placed great emphasis to sepsis and its definition [1]. The Third International Consensus Definitions for Sepsis and Septic Shock (Sepsis-3) revised the definitions emphasizing the role of the host response and the related pathophysiological mechanisms inducing organ dysfunction [2]. The change of perspective from invading pathogens to the host response has radically transformed the vision of sepsis pathobiology in the last decades. The current concepts indicate that sepsis progress on a double track sustained by products of infecting microorganisms and by endogenous mediators derived from complement activation and by specific cell-surface receptors expressed on immune, epithelial, and endothelial cells. A complex system of intracellular signals is created by the binding of pathogen-associated molecular patterns (PAMPs) and damage-associated molecular patterns (DAMPs) that lead to the expression of several common gene classes involved in inflammation, adaptive immunity, and cellular metabolism [3]. Specifically, the recognition of PAMPs and DAMPs produces the recruitment of proinflammatory intermediates that initiate the expression of early activation genes [4].

## 2. Sepsis Related Immune-Paralysis

Recent data on septic patients clarified that host response may be hyper- or hyporeactive with an overwhelming inflammation associated with a boost of proinflammatory cytokines in the former and an immune-paralysis with the prevalence of anti-inflammatory cytokines and cellular apoptosis in the latter. Although proinflammatory and anti-inflammatory responses occur simultaneously, early phases of sepsis are usually characterized by hyperinflammatory processes associated with classical clinical signs ranging from slight to severe impairment of organ function, including shock appearance [5]. On the other hand, immune-suppressive state becomes predominant in later stages of sepsis producing the so-called persistent inflammation/immunosuppression and catabolism syndrome (PICS) [6]. The hypothesis for explaining PICS developing are mainly two: (i) a persistent and dysregulated activity of PAMPs, DAMPs, inflammasomes, and tissue “alarmins” and (ii) the role of opportunistic infections (e.g., viral reactivation, infection* Acinetobacter* spp), changes in the host microbiota, and invasive procedures performed in critically ill patients [4]. Sepsis related immunosuppression causes profound changes in both the innate and adaptive immunity [7, 8] with persistent lymphopenia and high level of immature forms of myeloid cells. The sepsis induced immune dysfunction makes critically ill patient highly susceptible to colonization and secondary infections, including breakthrough infections, by opportunistic nosocomial multidrug resistant (MDR) bacteria. Therefore, patients carrying MDR bacteria might be considered a special population requiring specific strategies directed to supported immune system beyond the appropriate antibiotic therapy and standard supportive treatments.

## 3. Patients with MDR Bacteria: Why Are They a Special Population?

Sepsis and septic shock related to MDR bacteria are progressively increasing in the last decades with gram negative pathogens responsible for the majority of cases [9]. International guidelines define MDR bacteria as microorganisms nonsusceptible in vitro to at least three different antimicrobial categories (previously excluding intrinsic resistance), XDR as nonsusceptible to at least one agent in all but two or fewer antimicrobial categories, and PDR as resistant to any agents in all antimicrobial classes tested [10]. The burden of infections sustained by MDR bacteria is variable in different areas: world data show a lower incidence in northwest of Europe, USA, and Canada and a higher incidence in southeast of Europe, Latin America, and Asia Pacific [11]. According to recent studies, around one-third of the intensive care unit (ICU) acquired infections are sustained by MDR bacteria most of whom are* Acinetobacter* spp.,* Klebsiella pneumoniae*, and* Pseudomonas* spp. isolates [12]. The capability of these bacteria to survive for prolonged time in the hospital environment, the high risk of transfer among patients and healthcare staff, and the antibiotic resistance are responsible for their increasing widespread. Note that MDR strains are progressively increasing also in community acquired infections and the acquisition of these pathogens through travels in different world regions is becoming frequent.

MDR infections influences patients' outcome with higher mortality rates in metallo-*β*-lactamases* Enterobacteriaceae* and* Pseudomonas aeruginosa* and in carbapenem-resistant* Klebsiella pneumoniae*, likely due to the delay in the appropriate antimicrobial therapy [13]. The Centre for control of Diseases calculates that gram negative MDR infections are responsible for approximately 40,000 cases and more than 2,800 deaths in the United States (CDC 2013 Threat report). It is well known that the administration of an appropriate antimicrobial therapy within the first hour of diagnosis is strongly recommended in the management of patients septic shock and that an initial noneffective therapy is related to increased mortality [14]. In MDR infections the choice of an appropriate antimicrobial treatment is more complicated. In patients with bloodstream infections sustained by ESBL producing microorganisms, an appropriate antibiotic therapy reduced the 3-week mortality by 40% compared to a nonappropriate one [15].

The risk factors for acquisition of MDR infections are well known and related to both specific characteristics of patients and local epidemiology. The former includes specific conditions such as advanced age, diabetes, end-stage liver disease, immunosuppressive therapy, use of corticosteroids, malignancy, organ transplantation, recent surgery, recent exposure (<3 months) to antibiotic therapy, prior hospital admission, and MDR colonization [16]. It is noteworthy that the majority of these factors are related to a possible dysfunction of the immune response. In fact, advanced age is associated with a progressive dysfunction of immune system, both cell-mediated and humoral, defined as immunosenescence [17–19]. Similarly, in patients with malignancies, the growth of tumors takes place developing a condition of immunotolerance which allows cancer cells to escape from elimination [20, 21]. Tumors create an immunosuppressive microenvironment by producing mediators (IL-10, TGF-*β*, and VEGF) responsible for maturation and expansion of suppressive immune cells such as regulatory T cells, immature dendritic cells, and tumor associated macrophages [22]. Due to their immunocompromised condition, also cirrhotic patients have a high risk of developing infections. In these patients the magnitude of cellular immune depression is comparable to patients with severe sepsis with defects in innate immunity caused by a persistent activation of compensatory anti-inflammatory mechanisms to counteract the high burden of proinflammatory mediators occurring in end-stage liver disease [23, 24].

The relationship between MDR infections and the host immune response is so far unclear. A recent study described the interactions between different clones and resistance phenotypes of* Klebsiella pneumoniae* and innate immune response. In vitro stimulation of human peripheral blood mononuclear cells (PBMCs) with different heat-killed isolates of* K. pneumoniae* led to different patterns of TNF-*α* production. In particular, the highly virulent KPC-producing isolates of the ST17 clones are associated with low release of both TNF-*α* and IL-17 mediated by toll like receptor 9 that may contribute to a state of immunosuppression [25]. A similar work on* P. aeruginosa* showed that antibiotic susceptible isolates induce a significantly higher production of IL-1*β* and IL-6 and by human monocytes compared to MDR ones [26]. These results suggest that multidrug resistance could play a role in the modulation of host both innate and adaptive immune response. However, further studies are needed to better understand these complex relationships and the potential relevance of a specific immunomodulatory therapy in these infections.

## 4. The Role of Immune Adjuvant Therapies in MDR Infections

As described above, immune-paralysis is a hallmark of patients colonized or infected by MDR bacteria and, therefore, the development of treatments directed to restore the function of immune response may be useful to support antibiotic therapy in this specific population. Although many immune therapies have been investigated on animal models of sepsis, only few of them have been properly evaluated in patients (Table 1).

### 4.1. Extracorporeal Blood Purification Techniques

Different extracorporeal blood purification techniques have been recently developed and used during sepsis to remove endotoxins and proinflammatory mediators that play a substantial role in the inflammatory response of the host. Unfortunately, the effects of these treatments on the patient's immune response are unknown, particularly the long-term effects on the mechanisms leading to immune-paralysis. As regards impact on clinical outcome, two meta-analyses showed no benefits by the use of high volume hemofiltration [27, 28] as well as of cascade hemofiltration, despite promising results in an animal model [29, 30]. The use of hemoperfusion with polymyxin-B (PMX-B) for endotoxin blood removal showed contrasting results but a potential benefit in patients after emergency abdominal surgery seems to be plausible [31–33]. The just completed EUPHRATES trial will clarify better the role of this technique in septic patients with endotoxemia [34]. Interestingly, beyond endotoxin removal, immune-modulatory effects of PMX-B hemoperfusion has been also postulated with possible positive effects on the prevention of immune-paralysis [35]. Similarly, the association between plasma filtration and adsorption (CPFA) seems to have positive effects on the immune response with an increase in HLA-DR expression on monocytes and a restored lipopolysaccharide induced TNF-*α* production [36]. Unfortunately, a recent randomized control trial in patients with septic shock did not show significant benefits by the use of CPFA [37]. Highly adsorptive membranes and high cut-off membranes can also be used to obtain a blood purification and the progressive optimization of these techniques will lead to preservation of useful molecules and a more selective removal of inflammatory mediators [38]. However, so far evidences are only anecdotal and they should be used only for research purposes or compassionate use.

### 4.2. Pharmacological Approaches

The use of different molecules able to modulate the immune system has been also proposed in septic patients. Granulocytes-macrophage colony stimulating factor (GM-CSF) and interferon-*γ* (INF-*γ*) have been tested because of their effects on antigen presenting cells whose function in septic shock is deeply impaired. A meta-analysis of randomized trials in septic shock patients showed a better infection clearance in treated patients but no improvement in 28-day mortality [39]. It is notable that when GM-CSF was administered in patients with immune dysfunction (i.e., low mHLA-DR expression), its use was associated with prompt restoring of immune functioning and to reduced mechanical ventilation time and hospital length of stay [40]. INF-*γ* has been also administrated in subjects with trauma and burns with contradictory results. Again, it is to underline that, in burn patients with significant reduction of HLA-DR expression on monocytes, its use concomitant to GM-CSF was able to increase HLA-DR and to restore TNF-*α* secretion in ex vivo stimulated PBMCs [41].

Another potential target is the PD-1/PD-L pathway. Septic patients show an increased expression of PD-1 on T cells which leads to inhibition of cell proliferation, induction of IL-10 secretion, apoptosis, and anergy. Different studies have observed that block of this axis is able to improve survival in murine models of sepsis. In human antibodies anti-PD-1 and anti PD-L have been tested only to treat different types of cancer inducing a restoration of T cell activity [42]. In septic shock the PD-1 expression on T cells and/or PD-L expression on antigen presenting cells could be used as biomarkers of T cell exhaustion to drive anti-PD-1 and anti PD-L antibody administration. Other inhibitory receptors on T cell surface, such as TIM-3, LAG-3, CTLA-4, and BTLA, could be used for the same purpose in sepsis and septic shock but clinical trials are still lacking [43].

The use of recombinant interleukins in order to improve lymphocytes survival and function has been only experimented in HIV and cancer patients, but the potential benefits of these pleiotropic molecules have been demonstrated in animal models of septic shock. IL-7 has an antiapoptotic effect on T cell and is a crucial factor for lymphocyte production, maturation, and proliferation [44]. In different murine models of sepsis, the use of IL-7 is able to restore depleted T cells in lymphoid organs and induce T cell proliferation and INF-*γ* secretion leading to a significant improvement in survival [45]. IL-15 appears also as an interesting option because it promotes survival of dendritic cells and contributes to natural killer-dendritic cell interactions combined with antiapoptotic and function-enhancing properties on lymphocytes [46].

Compared to the above therapies, more data exists on the effects of intravenous immunoglobulins (IVIG) administration. Two preparations obtained from plasma of healthy donors are available: polyclonal standard IgG and IgM-enriched preparation. Both preparations are able to determine pathogen clearance but the higher killing on gram negative bacteria is obtained with IgM preparation because of specific proprieties of IgM fraction in neutralization and clearance of toxins [47, 48]. Beyond pathogen and toxin clearance, the pleiotropic effects on the immune response of endogenous immunoglobulins combined to the reduction of circulating IgG and IgM in nonsurvivors make the use of IVIG in patients with sepsis attractive [49, 50]. A recent meta-analysis of 18 trials in septic patients reveals a reduction in mortality using IVIG compared to control arm, in particular by using IgM-enriched formulation [49]. However, the heterogeneity in terms of type of preparation, dosage, and duration hinders the significance of the positive results observed. Unfortunately, in IVIG studies, neither patient's Ig plasma concentration nor other immunological markers have been ever used to identify patient at risk for immune failure. The use of specific biomarkers to identify patient who could benefit from immune therapy appears to be fundamental. In this light, the measurement of immunoglobulin plasma levels and their kinetic may be considered an appropriate guide to decide the use and to titrate the dose of IVIG [51].

## 5. Conclusions

The management of septic shock in patients suffering from infection caused by MDR/XDR bacteria is a true challenge. Taking into account the fact that multiresistant microorganisms are spreading worldwide, the probability to face such a patient is no longer an extraordinary event. The application of the Surviving Sepsis Campaign guidelines [52] and timely administration of antibiotics are often ineffective in these patients due to their frailty and poor immunological status [41]. Therefore, the use of adjunctive therapies for restoring immune function seems to be very promising but, unfortunately, sound evidences are not yet available. Waiting for the results of the ongoing trials, we believe that in patients with sepsis by MDR/XDR infections the capability of immune response should be carefully monitored by appropriate biomarkers.

## Figures and Tables

**Table 1:** The rationale and the possible adverse reactions of adjunctive immune-modulatory therapies in patients with sepsis.

	Potential advantages	Potential adverse effects
Extracorporeal blood purification techniques	(i) Removal of endotoxin (ii) Removal of middle molecular weight molecules (e.g., cytokines) (iii) Increase in HLA-DR expression on monocytes (iv) Restoration of TNF-*α* production	(i) Decrease of blood pressure (ii) Bleeding due to anticoagulant use (iii) Removal of drugs (e.g., amines, antibiotics) (iv) Possible removal of useful molecules (e.g., immune mediators)

Granulocytes-macrophage colony stimulating factor Interferon-*γ*	(i) Proliferation and maturation of granulocyte and monocyte precursor cells (ii) Stimulation of antigen presenting cells (iii) Increase in mHLA-DR expression (iv) Production of proinflammatory cytokines	(i) Fever (ii) Headache (iii) Edema (iv) Bone pain (v) Shortness of breath

PD-1/PD-L pathway	(i) Antiapoptotic effect (ii) Blockade of negative regulatory molecules	(i) Rare autoimmune reactions for long-term administration

Interleukin-7	(i) Stimulation of proliferation, maturation, and survival of T cells (ii) Increase of TCR repertoire diversity (iii) Production of proinflammatory cytokines	(i) Rare induction of fever and capillary leak syndrome

Interleukin-15	(i) Antiapoptotic effect on T cells and NK cells (ii) Expansion and activation of NK cells and CD8 memory T cells (iii) Stimulation of NK cells-dendritic cells crosstalk (iv) Antiapoptotic effect on dendritic cells (v) Production of proinflammatory cytokines	(i) Fever (ii) Rigor (iii) Hypotension (iv) Capillary leak syndrome (v) Nausea

Intravenous immunoglobulins	(i) Pathogen and apoptotic cells clearance (ii) Scavenging of toxins and mediators (iii) Anti-inflammatory effects (iv) Antiapoptotic effects on immune cells	(i) Rare allergic reactions

## References

[1] Bone R. C., Sibbald W. J., Sprung C. L. (1992). The ACCP-SCCM consensus conference on sepsis and organ failure. *Chest*.

[2] Seymour C. W., Liu V. X., Iwashyna T. J. (2016). The third international consensus definitions for sepsis and septic shock (Sepsis-3). *The Journal of the American Medical Association*.

[3] Tang D., Kang R., Coyne C. B., Zeh H. J., Lotze M. T. (2012). PAMPs and DAMPs: signal 0s that spur autophagy and immunity. *Immunological Reviews*.

[4] Hotchkiss R. S., Moldawer L. L., Opal S. M., Reinhart K., Turnbull I. R., Vincent J. (2016). Sepsis and septic shock. *Nature Reviews Disease Primers*.

[5] Hotchkiss R. S., Monneret G., Payen D. (2013). Sepsis-induced immunosuppression: from cellular dysfunctions to immunotherapy. *Nature Reviews Immunology*.

[6] Mira J. C., Gentile L. F., Mathias B. J. (2016). Sepsis pathophysiology, chronic critical illness, and persistent inflammation-immunosuppression and catabolism syndrome. *Critical Care Medicine*.

[7] Drewry A., Samra N., Skrupky L., Fuller B., Compton S., Hotchkiss R. (2014). Persistent lymphopenia after diagnosis of sepsis predicts mortality. *Shock*.

[8] Delano M. J., Scumpia P. O., Weinstein J. S. (2007). MyD88-dependent expansion of an immature GR-1 +CD11b+ population induces T cell suppression and Th2 polarization in sepsis. *Journal of Experimental Medicine*.

[9] Cornaglia G., Giamarellou H., Rossolini G. M. (2011). Metallo-*β*-lactamases: a last frontier for *β*-lactams?. *The Lancet Infectious Diseases*.

[10] Magiorakos A.-P., Srinivasan A., Carey R. B. (2012). Multidrug-resistant, extensively drug-resistant and pandrug-resistant bacteria: an international expert proposal for interim standard definitions for acquired resistance. *Clinical Microbiology and Infection*.

[11] Jones R. N. (2001). Resistance patterns among nosocomial pathogens: trends over the past few years. *Chest*.

[12] Tabah A., Koulenti D., Laupland K. (2012). Characteristics and determinants of outcome of hospital-acquired bloodstream infections in intensive care units: the EUROBACT international cohort study. *Intensive Care Medicine*.

[13] Gupta N., Limbago B. M., Patel J. B., Kallen A. J. (2011). Carbapenem-resistant enterobacteriaceae: epidemiology and prevention. *Clinical Infectious Diseases*.

[14] Ferrer R., Artigas A., Suarez D. (2009). Effectiveness of treatments for severe sepsis: a prospective, multicenter, observational study. *American Journal of Respiratory and Critical Care Medicine*.

[15] Tumbarello M., Sanguinetti M., Montuori E. (2007). Predictors of mortality in patients with bloodstream infections caused by extended-spectrum-beta-lactamase-producing Enterobacteriaceae: importance of inadequate initial antimicrobial treatment. *Antimicrob Agents Chemother*.

[16] Bassetti M., Carnelutti A., Peghin M. (2016). Patient specific risk stratification for antimicrobial resistance and possible treatment strategies in gram-negative bacterial infections. *Expert Review of Anti-infective Therapy*.

[17] Weiskopf D., Weinberger B., Grubeck-Loebenstein B. (2009). The aging of the immune system. *Transplant International*.

[18] Chen J. (2004). Senescence and functional failure in hematopoietic stem cells. *Experimental Hematology*.

[19] Ginaldi L., De Martinis M., D'Ostilio A., Marini L., Loreto M. F., Quaglino D. (1999). The immune system in the elderly: III. Innate immunity. *Immunologic Research*.

[20] Nurieva R., Wang J., Sahoo A. (2013). T-cell tolerance in cancer. *Immunotherapy*.

[21] Marincola F. M., Jaffee E. M., Hicklin D. J., Ferrone S. (1999). Escape of human solid tumors from T-cell recognition: molecular mechanisms and functional significance. *Advances in Immunology*.

[22] Gabrilovich D. (2004). Mechanisms and functional significance of tumour-induced dendritic-cell defects. *Nature Reviews Immunology*.

[23] Wasmuth H. E., Kunz D., Yagmur E. (2005). Patients with acute on chronic liver failure display ‘sepsis-like’ immune paralysis. *Journal of Hepatology*.

[24] Antoniades C. G., Berry P. A., Wendon J. A., Vergani D. (2008). The importance of immune dysfunction in determining outcome in acute liver failure. *Journal of Hepatology*.

[25] Pantelidou I.-M., Galani I., Georgitsi M., Daikos G. L., Giamarellos-Bourboulis E. J. (2015). Interactions of klebsiella pneumoniae with the innate immune system vary in relation to clone and resistance phenotype. *Antimicrobial Agents and Chemotherapy*.

[26] Giamarellos-Bourboulis E. J., Plachouras D., Tzivra A. (2004). Stimulation of innate immunity by susceptible and multidrug-resistant Pseudomonas aeruginosa: An in vitro and in vivo study. *Clinical and Experimental Immunology*.

[27] Clark E., Molnar A. O., Joannes-Boyau O., Honoré P. M., Sikora L., Bagshaw S. M. (2014). High-volume hemofiltration for septic acute kidney injury: a systematic review and meta-analysis. *Critical Care*.

[28] Borthwick E. M. J., Hill C. J., Rabindranath K. S., Maxwell A. P., McAuley D. F., Blackwood B. (2013). High-volume haemofiltration for sepsis. *Cochrane Database of Systematic Reviews*.

[29] Rimmele T., Hayi-Slayman D., Page M., Rada H., Monchi M., Allaouchiche B. (2009). Cascade hemofiltration: principle, first experimental data. *Annales Francaises D’Anesthesie Et De Reanimation*.

[30] Quenot J.-P., Binquet C., Vinsonneau C. (2015). Very high volume hemofiltration with the Cascade system in septic shock patients. *Intensive Care Medicine*.

[31] Zhou F., Peng Z., Murugan R., Kellum J. A. (2013). Blood purification and mortality in sepsis: a meta-analysis of randomized trials. *Critical Care Medicine*.

[32] Payen D. M., Guilhot J., Launey Y. (2015). Early use of polymyxin B hemoperfusion in patients with septic shock due to peritonitis: a multicenter randomized control trial. *Intensive Care Medicine*.

[33] Cruz D. N., Antonelli M., Fumagalli R. (2009). Early use of polymyxin B hemoperfusion in abdominal septic shock: the EUPHAS randomized controlled trial. *the Journal of the American Medical Association*.

[34] Klein D. J., Foster D., Schorr C. A., Kazempour K., Walker P. M., Dellinger R. P. (2014). The EUPHRATES trial (evaluating the use of polymyxin B hemoperfusion in a randomized controlled trial of adults treated for endotoxemia and septic shock): study protocol for a randomized controlled trial. *Trials*.

[35] Esteban E., Ferrer R., Alsina L., Artigas A. (2013). Immunomodulation in sepsis: the role of endotoxin removal by polymyxin B-immobilized cartridge. *Mediators of Inflammation*.

[36] Mao H., Yu S., Yu X. (2009). Effect of coupled plasma filtration adsorption on endothelial cell function in patients with multiple organ dysfunction syndrome. *The International Journal of Artificial Organs*.

[37] Livigni S., Bertolini G., Rossi C. (2014). Efficacy of coupled plasma filtration adsorption (CPFA) in patients with septic shock: a multicenter randomised controlled clinical trial. *BMJ Open*.

[38] Haase M., Bellomo R., Baldwin I. (2007). Hemodialysis membrane with a high-molecular-weight cutoff and cytokine levels in sepsis complicated by acute renal failure: a phase 1 randomized trial. *American Journal of Kidney Diseases*.

[39] Bo L., Wang F., Zhu J., Li J., Deng X. (2011). Granulocyte-colony stimulating factor (G-CSF) and granulocyte-macrophage colony stimulating factor (GM-CSF) for sepsis: a meta-analysis. *Critical Care*.

[40] Meisel C., Schefold J. C., Pschowski R. (2009). Granulocyte-macrophage colony-stimulating factor to reverse sepsis-associated immunosuppression: a double-blind, randomized, placebo-controlled multicenter trial. *American Journal of Respiratory and Critical Care Medicine*.

[41] Döcke W., Randow F., Syrbe U. (1997). Monocyte deactivation in septic patients: restoration by IFN-*γ* treatment. *Nature Medicine*.

[42] Topalian S. L., Hodi F. S., Brahmer J. R. (2012). Safety, activity, and immune correlates of anti-PD-1 antibody in cancer. *The New England Journal of Medicine*.

[43] Sierro S., Romero P., Speiser D. E. (2011). The CD4-like molecule LAG-3, biology and therapeutic applications. *Expert Opinion on Therapeutic Targets*.

[44] Ma A., Koka R., Burkett P. (2006). Diverse functions of IL-2, IL-15, and IL-7 in lymphoid homeostasis. *Annual Review of Immunology*.

[45] Kasten K. R., Prakash P. S., Unsinger J. (2010). Interleukin-7 (IL-7) treatment accelerates neutrophil recruitment through *γδ* T-cell IL-17 production in a murine model of sepsis. *Infection and Immunity*.

[46] Hiromatsu T., Yajima T., Matsuguchi T. (2003). Overexpression of interleukin-15 protects against Escherichia coli-induced shock accompanied by inhibition of tumor necrosis factor-*α*-induced apoptosis. *Journal of Infectious Diseases*.

[47] Rossmann F. S., Kropec A., Laverde D., Saaverda F. R., Wobser D., Huebner J. (2015). In vitro and in vivo activity of hyperimmune globulin preparations against multiresistant nosocomial pathogens. *Infection*.

[48] Trautmann M., Held T. K., Susa M. (1998). Bacterial lipopolysaccharide (LPS)-specific antibodies in commercial human immunoglobulin preparations: Superior antibody content of an IgM- enriched product. *Clinical and Experimental Immunology*.

[49] Busani S., Damiani E., Cavazzuti I., Donati A., Girardis M. (2016). Intravenous immunoglobulin in septic shock: review of the mechanisms of action and meta-analysis of the clinical effectiveness. *Minerva Anestesiologica*.

[50] Bermejo-Martín J. F., Rodriguez-Fernandez A., Herrán-Monge R. (2014). Immunoglobulins IgG1, IgM and IgA: A synergistic team influencing survival in sepsis. *Journal of Internal Medicine*.

[51] Dietz S., Lautenschläger C., Müller-Werdan U. (2016). Serum IgG levels and mortality in patients with severe sepsis and septic shock: The SBITS data. *Medizinische Klinik Intensivmedizin und Notfallmedizin*.

[52] Dellinger R. P., Levy M. M., Rhodes A. (2017). Surviving Sepsis Campaign: International Guidelines for Management of Sepsis and Septic Shock: 2016. *Intensive Care Medicine*.

